# Role of the Triceps Surae Muscles in Patients Undergoing Anterior Cruciate Ligament Reconstruction: A Matched Case-Control Study

**DOI:** 10.3390/jcm9103215

**Published:** 2020-10-07

**Authors:** Hye Chang Rhim, Jin Hyuck Lee, Seung-Beom Han, Kyun-Ho Shin, Dong Won Suh, Ki-Mo Jang

**Affiliations:** 1 Department of Orthopaedic Surgery, Anam Hospital, Korea University College of Medicine, Seoul 02841, Korea; hr233@cornell.edu (H.C.R.); sbhan1107@gmail.com (S.-B.H.); Kyunho.shin@gmail.com (K.-H.S.); 2 Department of Sports Medical Center, Anam Hospital, Korea University College of Medicine, Seoul 02841, Korea; gnkfccc@hanmail.net; 3 Department of Orthopaedic Surgery, Barunsesang Hospital, Seongnam 13497, Korea; s9187@hanmail.net

**Keywords:** anterior cruciate ligament reconstruction, muscle reaction time, muscle strength, triceps surae

## Abstract

A limited number of studies has investigated the gastrocnemius and soleus in patients undergoing anterior cruciate ligament reconstruction (ACLR). This study investigated the muscle strength (Nm kg^−1^ × 100) and reaction time (acceleration time (AT), milliseconds) of thigh and calf muscles in patients undergoing ACLR. Thirty-two patients with ACLR and 32 normal control subjects were included. One year postoperatively, the strength of thigh muscles was significantly reduced after ACLR compared with that of controls (hamstring: 80 ± 31.3 vs. 142 ± 26.4, *p* < 0.001, quadriceps: 159 ± 63.7 vs. 238 ± 35.3, *p* < 0.001). However, the strength of calf muscles was not significantly different compared with that of controls (gastrocnemius: 77 ± 22.9 vs. 81 ± 22.5, *p* = 0.425, soleus: 54 ± 15.9 vs. 47 ± 16.1, *p* = 0.109). The AT of calf muscles was significantly faster after ACLR than that of controls (gastrocnemius: 26 ± 9.8 vs. 31 ± 9, *p =* 0.030, soleus: 18 ± 6.7 vs. 22 ± 8.5, *p =* 0.026). The AT of thigh muscles was significantly elongated after ACLR than that of controls (hamstring: 72 ± 18 vs. 55 ± 12.4, *p* < 0.001, quadriceps: 63 ± 17.6 vs. 47 ± 17, *p* < 0.000). The strength of thigh muscles was reduced, and the ATs of thigh muscles were slower one year after ACLR. However, the AT of the triceps surae was faster than that of controls. This may implicate a compensatory mechanism of the triceps surae for the weakness and delayed activation in hamstring and quadriceps muscles.

## 1. Introduction

The anterior cruciate ligament (ACL) is one of the most commonly injured structures in the knee [1]. ACL injuries are sustained because of side cutting maneuvers or when landing from a jump [2]. In order to prevent such injuries, the knee joint needs to be stabilized and protected from excessive loads on the ligaments. Lower limb muscle strength and neuromuscular control are important factors in the stabilization of the knee joint [2,3].

Muscles around the knee such as the quadriceps, hamstrings, and triceps surae serve as either agonists or antagonists of the ACL in achieving the joint stability. The quadricep muscles are antagonists of the ACL; they induce anterior translation of the tibia and increase ACL load. The hamstring muscles act as agonists of the ACL; they generate a flexion moment and limit tibial anterior translation [4,5,6]. While the influence of quadricep and hamstring muscles on the ACL is well documented, the role of the triceps surae, composed of gastrocnemius and soleus, remains less explored and controversial.

In vivo measurements of ACL strain [7] and a computational model using in vivo data [8] showed that gastrocnemius may act as an antagonist of the ACL. In contrast, another model found that gastrocnemius plays a protective role in reducing ACL load [9]. Increased gastrocnemius forces corresponded to lower ACL loads during single-leg jump landings [10]. Unlike gastrocnemius, soleus does not cross the knee joint, however, previous studies suggested that soleus may act as an agonist of ACL by resisting forward rotation of the tibia around the ankle joint [2,11]. However, a limited number of studies investigated the role of soleus, and no study to date has evaluated the muscle strength and reaction time of gastrocnemius and soleus in patients treated with ACL reconstruction (ACLR) compared with normal control.

Therefore, the purpose of this study was to measure the muscle strength and the reaction time of the triceps surae, quadriceps, and hamstrings in patients who have undergone ACLR using hamstring tendon autografts compared with those of normal control subjects. It was hypothesized that muscle strength or reaction time of the triceps surae in the operated knee would be stronger or faster than those of the control in order to compensate for deficient quadriceps and hamstrings strength before and after ACLR.

## 2. Materials and Methods

### 2.1. Patient Enrollment

This study complied with the Declaration of Helsinki and was approved by the institutional review board at our institute (IRB No. 2017AN0178). We retrospectively reviewed the strength and reaction time of thigh and calf muscles in 269 patients who underwent primary ACLR with hamstring autografts at our institute from 2013 to 2017. The operations were performed by two knee arthroscopic specialists. Among the patients, those who completed isokinetic tests for both thigh and calf muscles preoperatively and one year postoperatively were included. We excluded 237 patients based on the following criteria: bilateral ACL injury, ACLR using allograft, concomitant injuries such as meniscus tear or collateral ligaments, and professional athletic patients. We excluded these patients because bilateral ACL injury or concomitant meniscus tear or collateral ligament injuries themselves can affect the clinical outcomes after ACLR [12,13]. Therefore, we only included the patients with isolated ACL injuries in order to investigate the function of calf and thigh muscles that were not affected by the concomitant injuries. We also excluded patients with insufficient isokinetic muscle test data. We used the coefficient of variation (CV) to determine the reliability of isokinetic muscle test data because it assesses the stability of measurements across repeated trials. Previous studies reported that the acceptable ranges of CV were 4.0–19.9 for plantar flexion, 3.9–16.5 for knee flexion, and 4.9–8.1 for knee extension [14,15]. If the CVs were not in the acceptable ranges for each isokinetic test, the data were considered insufficient. After exclusion, 32 patients were finally enrolled in the present study. The enrolled patients were not treated with preoperative rehabilitation, but patient education was performed regarding importance and general protocol of postoperative rehabilitation before surgery. Muscle strength and acceleration time (AT) were assessed routinely on the day before surgery and one year postoperatively.

To confirm the differences in muscle strength and reaction time, individual matching for the normal control group was performed from the database, according to age, body mass index (BMI), sex, and sport activity level. A previous study categorized sports activity levels into four levels according to frequency and participation in sports that involve jumping, hard pivoting, and cutting. Using this study as guidance, we categorized patients into the “high” category if they were involved in such sports one day or more per week and into the “low” category if they were involved in such sports 1–3 times a month or less [16]. The sports activity level was assessed because potential differences in the level may affect the clinical outcomes. If there were no perfect matching cases using these factors, controls with the closest values of age, BMI, and sex were selected in sequence. As a result, 32 normal subjects were selected as a control group from our database of volunteers without history of pain, injuries, and previous surgeries in the knee and ankle joints who agreed to participate in this study. Normal control subjects were included because the non-operated knees of patients with ACL injury may be affected by injury and reconstruction processes [3,17].

### 2.2. Surgical Technique

Each patient was given a spinal or general anesthetic and positioned supine. A tourniquet was applied on the proximal thigh and the limb was supported by a leg holder with the knee at 90° of flexion. Standard knee arthroscopy was performed with anterolateral and anteromedial portals using a 4.0 mm arthroscope. After the joint was fully examined, an approximately 3cm oblique skin incision was made anteromedially to the tibial tuberosity to harvest the semitendinosus and gracilis tendons, which were harvested with a standard tendon stripper. Both tendons were prepared on a back table, and the ends of the graft was sutured to form a closed loop. Then, the femoral and tibial tunnels were made. After a central transpatellar tendon portal was made, the femoral tunnel was created using the ACL RetroConstruction System and FlipCutter drill (Arthrex, Naples, FL, US) through the anatomic outside-in retrograde-reaming technique [18]. Then, the tibial tunnel was made with the use of an ACL tibial guide. Finally, the prepared graft was fixed by a bioabsorbable interference screw with a post tie for the tibial side and a TightRope^®^ RT (Arthrex, Naples, FL, US) for the femoral side [19]. The two surgeons performed all operations using the same technique.

### 2.3. Postoperative Rehabilitation

Postoperatively, ACL rehabilitation was based on the same protocol described in a previous study [3] and was divided into three phases. All patients were prescribed with the same one-hour exercise program to perform twice a week. The initial phase lasted up to postoperative 6 weeks, and the goal of this period was to gain range of motion (ROM), pain control, and normal gait. The second phase lasted up to postoperative 12 weeks and was intended to improve strength for core, knee, and hip muscles by performing bridge, squat, and leg press exercises and to improve proprioception and neuromuscular control by performing balance training such as star-excursion implanted with both eyes open and eyes closed. The final phase involved the period after postoperative 12 weeks and focused on enhancing functional performance. After postoperative 6 months, the authors allowed gradual participation in sports activities in all patients. However, the types of sports, frequency, and intensity were individualized according to each patient’s physical and psychological conditions assessed by instability tests, muscle strength tests, neuromuscular control tests, functional performance tests, self-reported outcome measures, and psychological evaluation. Generally, patients began to participate in non-contact sports activities such as running and jumping as tolerated and gradually moved onto contact sports under the guidance of the surgeon and physical therapists.

### 2.4. Assessment of Isokinetic Muscle Performances

#### 2.4.1. Triceps Surae Strength

**Figure 1:**
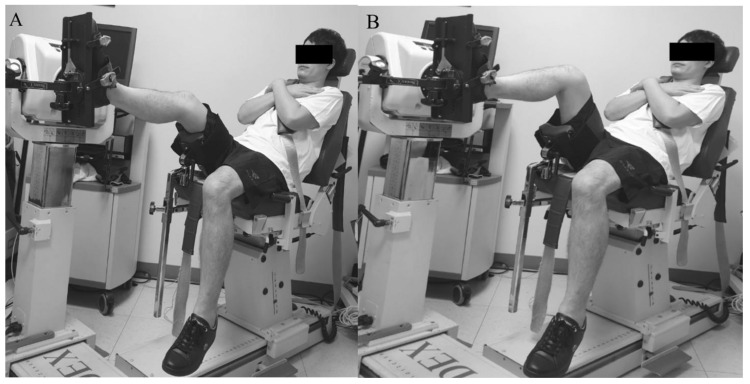
Measurement of gastrocnemius (**A**) and soleus (**B**) muscle strength.

Isokinetic triceps surae strength (concentric mode, Nm kg^−1^ × 100) was measured in a semi-seated position on a quantified isokinetic device (Biodex MultiJoint System 4, Biodex Medical Systems Inc., Shirley, NY) with 20° (Figure 1A) and 90° (Figure 1B) of knee flexion to evaluate the gastrocnemius [20] and soleus [21] strength, respectively. Each test consisted of five repetitions of plantar flexion for each leg (injured leg and uninjured leg, respectively) at 120°/sec, with a resting time of 30 s between the tests.

#### 2.4.2. Hamstring and Quadriceps Strength

**Figure 2:**
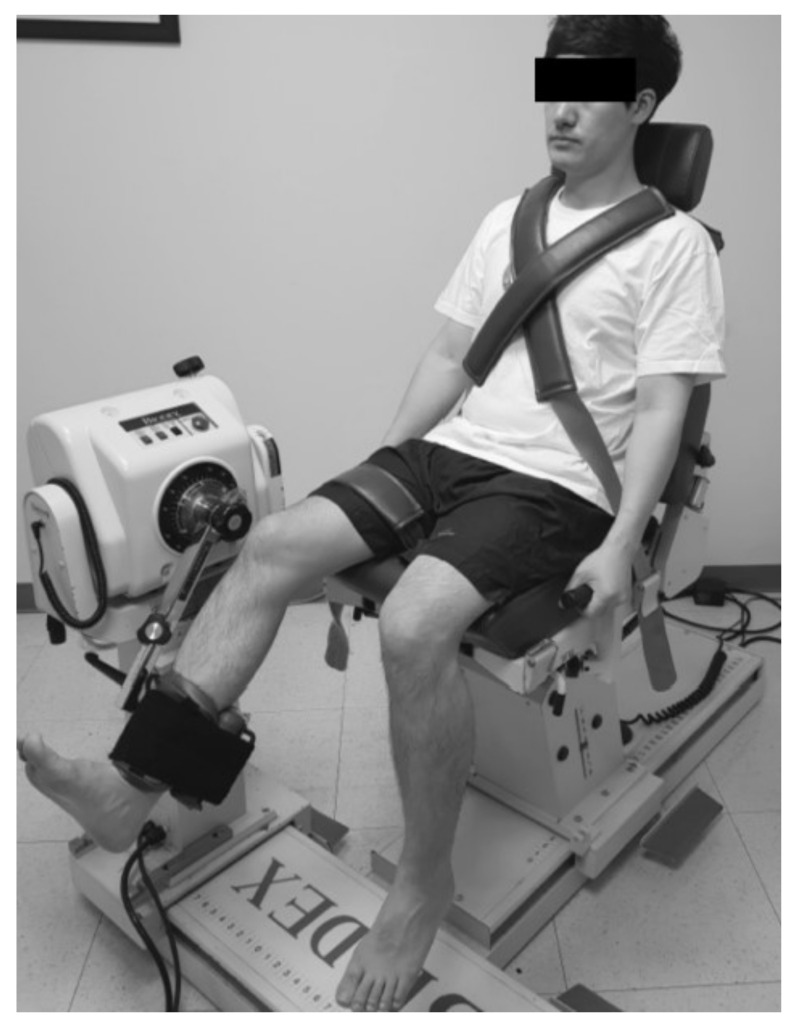
Measurement of hamstrings and quadriceps muscle strength.

The strength of isokinetic knee extension/flexion (concentric/concentric mode, Nm kg^−1^ × 100) was measured in a seated position on a quantified isokinetic device (Biodex MultiJoint System 4, Biodex Medical Systems Inc., Shirley, NY) with 90° flexion of the hip and knee joints. Each test consisted of 5 repetitions of extension/flexion (ROM, 90° to 0°) for each leg (injured leg and uninjured leg, respectively) at 180°/s with a resting time of 30 s between the tests. Flexor and extensor strengths were regarded as hamstring and quadriceps muscles strengths, respectively (Figure 2).

### 2.5. Assessment of Muscle Reaction Time (Acceleration Time)

A sufficient muscle reaction time is important in protecting joints against sports injuries that require rapid coordination of muscle reaction [22]. The mechanism of muscle reaction is related to the arthrokinetic reflex, which is affected by velocity and acceleration [23,24]. Previous studies used methodologies such as trapdoor experiments and electromyography, but isokinetic analysis is a more dynamic assessment method and simulates a behavior that is closer to the sport activity [25]. Therefore, in this study, muscle reaction time was measured based on the AT in the isokinetic strength test. The muscle reaction time (msec) was specifically measured for a pre-set angular velocity (180°/s for knee joint and 120°/s for ankle joint in our study) during maximal muscle contraction [3,24,26,27]. Higher values for AT suggested delayed muscle reaction. AT was calculated automatically using the Biodex advantage software.

### 2.6. Statistical Analysis

The sample size in the present study was based on a previous study investigating muscle strength in patients with lower extremity injury [28,29], and muscle strength difference >10% between the groups was considered significant. To determine the sample size, we conducted a priori power analysis with an alpha level of 0.05 and a power of 0.8. Effect size (Cohen’s d: 0.836) was calculated using the mean and standard deviation in a pilot study involving 5 knees in each group, which indicated that 24 knees in each group were required to adequately identify a clinically meaningful difference in muscle strength >10% between the groups. The power necessary to detect differences in muscle strength was 0.809.

The paired t-test was used to compare the muscle strengths and reaction times for gastrocnemius, soleus, hamstring, and quadricep muscles of both knees before and after ACLR. Student’s t-test was used to compare the muscle strength and reaction time for gastrocnemius, soleus, hamstring, and quadricep muscles of both knees between patients with ACLR and normal control subjects. To determine whether a continuous variable followed a normal distribution, the Shapiro test was used. Data were analyzed using the SPSS software version 17.0 (SPSS Inc., Chicago, IL, USA). A value of *p* < 0.05 was considered statistically significant.

## 3. Results

A total of 64 participants (32 patients vs. 32 normal controls) were enrolled in this study. Demographic data of the enrolled patients and normal control subjects are summarized in Table 1, and no differences were observed between the groups.

### 3.1. Isokinetic Muscle Strength

The strengths of gastrocnemius and soleus in the operated knees were significantly increased one year after ACLR compared with the strength before ACLR (gastrocnemius: 21 ± 13.9 vs. 77 ± 22.9, *p* = 0.001, soleus: 41 ± 10.9 vs. 54 ± 15.9, *p* < 0.001) (Table 2**)**. However, the strengths of gastrocnemius and soleus in the operated knees at postoperative one year was not significantly different compared with the strength of normal control subjects (gastrocnemius: 77 ± 22.9 vs. 81 ± 22.5, *p* = 0.425, soleus: 54 ± 15.9 vs. 47 ± 16.1, *p* = 0.109) (Table 3).

The strength of hamstrings and quadriceps in the operated knees was significantly greater one year after ACLR than the level before ACLR (hamstring: 49 ± 36.4 vs. 80 ± 31.3, *p* = 0.001, quadriceps: 98 ± 55.1 vs. 159 ± 63.7, *p* < 0.001) (Table 2). However, the strengths of hamstring and quadriceps in the operated knees one year after ACLR was significantly lower than in normal control subjects (hamstring: 80 ± 31.3 vs. 142 ± 26.4, *p* < 0.001, quadriceps: 159 ± 63.7 vs. 238 ± 35.3, *p* < 0.001) (Table 3).

In non-operated knees, the strengths of the gastrocnemius, soleus, hamstrings, and quadriceps were not significantly different between patients before and after ACLR (Table 2). Gastrocnemius and soleus strengths were not significantly different, but the strengths of hamstring and quadriceps were lower compared with those of control (Table 3).

### 3.2. Muscle Reaction Time (AT)

The ATs of gastrocnemius and soleus in the operated knees were not significantly different between patients before and after ACLR (*p* > 0.05) (Table 2). However, the ATs of gastrocnemius and soleus in the operated limbs one year after ACLR were significantly faster than in control limbs (gastrocnemius: 26 ± 9.8 vs. 31 ± 9, *p* = 0.030, soleus: 18 ± 6.7 vs. 22 ± 8.5, *p* = 0.026) (Table 3).

The ATs of hamstrings and quadriceps in the operated knees significantly improved one year after ACLR compared with the level before ACLR (hamstring: 89 ± 39.1 vs. 72 ± 18, *p* = 0.027, quadriceps: 94 ± 76.6 vs. 63 ± 17.6, *p* = 0.044) (Table 2). However, the ATs of hamstrings and quadriceps in the operated knees one year after ACLR were significantly slower than in normal control subjects (hamstring: 72 ± 18 vs. 55 ± 12.4, *p* < 0.001, quadriceps: 63 ± 17.6 vs. 47 ± 17, *p* < 0.001) (Table 3).

In non-operated knees, the ATs of gastrocnemius, soleus, hamstrings, and quadriceps were not significantly different between patients before and after ACLR compared with those of normal controls.

## 4. Discussion

To the best of our knowledge, this is the first study investigating muscle strength and reaction time of gastrocnemius and soleus of patients who have undergone ACLR compared with those of normal control subjects. The present study is unique in that the data were obtained pre-operatively in a temporary ACL-deficient state, and at one year after ACLR, which demonstrates patients’ adaptation to the new ACL following rehabilitation. The gastrocnemius and soleus in the operated knees after ACLR were as strong as those of controls while the strengths of quadriceps and hamstrings after ACLR were weaker than those of controls. Interestingly, the ATs of gastrocnemius and soleus after ACLR were significantly faster while the ATs of quadriceps and hamstrings after ACLR were slower compared with those of controls. This result may indicate that the muscle reaction ability of the triceps surae at one year after ACLR compensated for the weakness and delayed activation of quadriceps and hamstrings to increase joint stability.

### 4.1. Strength of Gastrocnemius and Soleus

In the present study, the strength of both gastrocnemius and soleus in the operated knees improved significantly at one year after ACLR and was not significantly different compared to the level of normal control subjects. A possible explanation for this improved strength of gastrocnemius derives from the increased mechanical advantage of the knee joint induced by changes in the tibial or femoral translation after ACLR. The moment arm of the plantar flexor muscles is affected by the flexion angle of knee joint [11]. At 20° of knee flexion after an ACL tear, the tibial anterior translation with respect to the femur may increase along with tensioning in the quadriceps muscle [11,30,31]. This phenomenon results in the progressive shortening of the moment arm of the gastrocnemius due to increased posterior subluxation of the femur. However, after ACLR, the posterior subluxation of the femur decreases, thereby increasing the moment arm and improving gastrocnemius strength. Further, while some studies suggested that gastrocnemius is an antagonist of the ACL [7,8,32], other studies found that gastrocnemius may have a protective role for the ACL [9,10,33]. Specifically, Morgan et al. showed that the gastrocnemius muscle compensated for decreased hamstring forces during the weight-acceptance phase of single-leg jump landing [10]. Since the patients included in our study manifested lower hamstring strength after ACLR, it is possible that the strength of gastrocnemius improved to compensate for weak hamstrings and protect a reconstructed ACL.

Despite limited research, soleus has been reported to be an agonist of the ACL [2,11]. In contrast to gastrocnemius, the strength of soleus is not affected by the tibial or femoral translation according to the angle of knee flexion because it does not cross the knee joint. Nevertheless, soleus was found to protect ACL in both static and dynamic settings by resisting tibial translation and exerting a posterior force on the tibia [2,11,33]. Our results showed that similar to gastrocnemius, the strength of soleus improved significantly after ACLR, close to that of normal control subjects. Even though the strength did not increase to greater than that of normal control subjects, a recovery to at least normal range might be necessary to further support the knee joint, especially when the strength of hamstrings are inadequate as seen in our patients.

### 4.2. Muscle Reaction Time

Reaction time is defined as the amount of time required to initiate motor response to a stimulus [34]. It is a component of neuromuscular control [35], and a faster muscle reaction time suggests earlier muscle recruitment for joint stability [36,37]. Knee joint stability is affected by co-activation and neuromuscular control of ligaments and muscles of the knee joint such as the ACL, quadriceps, hamstrings, and triceps surae [10,38,39]. Previous studies reported atrophy, weakness, and delayed muscle reaction time in the quadricep and hamstring muscles of the operated knees before and after ACLR [3,40,41]. After ACL injury and ACLR, however, co-contraction and rapid reaction of knee joint muscles is especially important in protecting the knee joint [30,31,42]. Klyne et al. found that there was rapid and prolonged muscle activation in the gastrocnemius muscle to increase knee joint stability in ACL-deficient knees compared to that of healthy subjects [42]. Consistent with this study, neuromuscular adaptation may occur even before ACLR, which represents a temporary ACL-deficient state, as the AT of the triceps surae was not significantly different between pre- and post-ACLR, but faster than that of controls. Furthermore, despite significant improvement in the ATs of hamstring and quadricep muscles after ACLR, they were still slower than those of controls. Therefore, the faster AT of the triceps surae after ACLR may represent a compensatory mechanism to increase joint stability [43,44,45] for the weakness and delayed muscle reaction times of hamstring and quadricep muscles. In addition, these faster ATs may partly explain the agonist role of both gastrocnemius and soleus muscles for the ACL.

### 4.3. Clinical Implications

Even though current evidence-based rehabilitation incorporates isometric and isotonic strength training of gastrocnemius and soleus during the early phase after ACLR [46], its importance may be underestimated by both clinicians and patients. The results of our study suggest that the lack of compensation, i.e., deficits in gastrocnemius and soleus muscle strengths or reaction times, may potentially increase the risk of joint instability and ACL re-injury. Therefore, the muscle strength of the triceps surae needs to be regularly evaluated and trained along with quadricep and hamstring muscles. In particular, strength training of soleus may be advantageous and important in ACL-deficient patients or patients after ACLR because its function is not affected by knee flexion angle. A stronger soleus may independently and partly resist tibial anterior translation regardless of co-contraction of the hamstrings and further enhances joint stability. Therefore, a strength training program targeting soleus may help prevent ACL injuries or minimize the risk graft rupture in patients undergoing ACLR.

### 4.4. Limitations

The present study had several limitations. First, due to the retrospective design, patients with missing data were excluded from the study. Further prospective studies are needed to elucidate the function of the triceps surae muscles more clearly after ACLR. Second, the ACLR was performed by two knee arthroscopic specialists. However, these two surgeons had equivalent technical knowledge and experience to carry out this procedure. They performed all operations using the same surgical technique (anatomical single bundle outside-in ACLR). Third, we could not assess the actual level of instability of the knee joint during isokinetic testing after ACLR. Kvist et al. reported that dynamic tibial translation during specific behavior is an important factor in knee functionality [47], and Patel et al. concluded that static tibial translation, such as that created with the anterior drawer test and Lachman test, was not associated with dynamic knee function [1]. Lastly, the central transpatellar tendon portal used during ACLR might have a negative impact on quadriceps strength and muscle reaction time. This portal was made for a better femoral footprint view and anatomic drilling during ACLR. In our cases, there were no patients who showed significant complications or complained of pain around the portal after the acute postoperative phase. Furthermore, a recent study demonstrated that the central transpatellar tendon portal during ACLR is safe and does not lead to significant clinical complications [48]. However, the effect of the central transpatellar portal on quadriceps function should be investigated further in future studies.

## 5. Conclusions

The strength of thigh muscles was reduced, and the ATs of thigh muscles were slower after ACLR. However, the strength of the triceps surae in patients after ACLR was not significantly different compared to that of normal controls, and the AT of the triceps surae was faster than that of controls. This adaptation may implicate a compensatory mechanism of gastrocnemius and soleus muscles for the weakness and delayed activation in hamstring and quadricep muscles. Rapid muscle recruitment may explain the protective role of the triceps surae in knee joint stability.

## Figures and Tables

**Table 1:** Demographic data of enrolled patients and normal control subjects.

	ACL Reconstruction (*n* = 32)	Normal Control (*n* = 32)	*p* Value
Gender (male/female)	12/20	15/17	0.448
Age (years) ^a^	30 ± 7 (23–44)	29 ± 8 (21–43)	0.384
Height (cm) ^a^	174 ± 7 (162–184)	173 ± 3 (157–187)	0.397
Weight (kg) ^a^	68 ± 10 (48–87)	69 ± 9 (54–84)	0.538
Body mass index (kg/m^2^) ^a^	22 ± 2 (17–29)	23 ± 1 (20–28)	0.217
Sports and activity (*n*, low:high)	13:40	9:31	0.736

ACL, anterior cruciate ligament; ^a^ The values are expressed as mean ± standard deviation. (minimum-maximum values).

**Table 2:** Muscle strength and acceleration time of both knees before and one year after anterior cruciate ligament (ACL) reconstruction of one knee.

	Operated Knees			Non-Operated Knees		
	Pre-OP	Post-OP 1 Year	p Value	Pre-OP	Post-OP 1 Year	p Value
Gastrocnemius strength	21 ± 13.9	77 ± 22.9	<0.001^a^	72 ± 22.2	78 ± 22.6	0.635
Soleus strength	41 ± 10.9	54 ± 15.9	<0.001^a^	50 ± 18.7	49 ± 15.0	0.866
Quadriceps strengths	98 ± 55.1	159 ± 63.7	<0.001^a^	198 ± 67.7	214 ± 54.1	0.245
Hamstring strengths	49 ± 36.4	80 ± 31.3	0.001 ^a^	90 ± 29.5	100 ± 25.9	0.112
Gastrocnemius AT	24 ± 10.8	26 ± 9.8	0.635	31 ± 11.3	29 ± 10.4	0.500
Soleus AT	22 ± 8.7	18 ± 6.7	0.070	23 ± 10.1	20 ± 7.1	0.244
Quadriceps AT	94 ± 76.6	63 ± 17.6	0.044 ^a^	61 ± 15.0	66 ± 54.9	0.555
Hamstrings AT	89 ± 39.1	72 ± 18	0.027 ^a^	69 ± 16.1	81 ± 75.2	0.355

ACL, anterior cruciate ligament; AT, acceleration time; OP, operation. Note: The values are expressed as mean ± standard deviation. Measurement units of knee muscle strength and neuromuscular control were Nm kg ^−1^ × 100 and millisecond, respectively. ^a^ Statistically significant.

**Table 3:** Muscle strength and acceleration time of both knees for patients having ACL reconstruction on one knee (postoperative one year) and normal control subjects.

	Operated Knees			Non-Operated Knees		
	ACL Reconstruction	Normal Control	p Value	ACL Reconstruction	Normal Control	p Value
Gastrocnemius strength	77 ± 22.9	81 ± 22.5	0.425	78 ± 22.6	81 ± 23.1	0.649
Soleus strength	54 ± 15.9	47 ± 16.1	0.109	49 ± 15.0	49 ± 15.9	0.967
Quadriceps strength	159 ± 63.7	238 ± 35.3	<0.001^a^	214 ± 54.1	247 ± 26.4	0.001 ^a^
Hamstrings strength	80 ± 31.3	142 ± 26.4	<0.001^a^	100 ± 25.9	168 ± 26.6	0.001 ^a^
Gastrocnemius AT	26 ± 9.8	31 ± 9	0.030 ^a^	29 ± 10.4	31 ± 7.3	0.273
Soleus AT	18 ± 6.7	22 ± 8.5	0.026 ^a^	20 ± 7.1	22 ± 8.6	0.109
Quadriceps AT	63 ± 17.6	47 ± 17	<0.001^a^	66 ± 54.9	42 ± 16	0.017
Hamstrings AT	72 ± 18	55 ± 12.4	<0.001^a^	81 ± 75.2	52 ± 15.8	0.033

ACL, anterior cruciate ligament; AT, acceleration time. Note: The values are expressed as mean ± standard deviation. Measurement units of knee muscle strength and neuromuscular control were Nm kg^−1^ × 100 and millisecond, respectively. ^a^ Statistically significant.
